# Understanding Patterns of Healthy Aging Among Men Who Have Sex With Men: Protocol for an Observational Cohort Study

**DOI:** 10.2196/25750

**Published:** 2021-09-23

**Authors:** James E Egan, Sabina A Haberlen, Steven Meanley, Deanna Ware, Andre L Brown, Daniel Siconolfi, Mark Brennan-Ing, Ron Stall, Michael W Plankey, M Reuel Friedman

**Affiliations:** 1 Department of Behavioral and Community Health Sciences Graduate School of Public Health University of Pittsburgh Pittsburgh, PA United States; 2 Center for LGBT Health Research Graduate School for Public Health University of Pittsburgh Pittsburgh, PA United States; 3 Department of Epidemiology Johns Hopkins University Baltimore, MD United States; 4 Department of Family and Community Health School of Nursing University of Pennsylvania Philadelphia, PA United States; 5 Department of Medicine, Division of General Internal Medicine Georgetown University Medical Center Washington, DC United States; 6 RAND Corporation Pittsburgh, PA United States; 7 Brookdale Center for Healthy Aging Hunter College New York, NY United States; 8 Department of Infectious Diseases and Microbiology Graduate School of Public Health University of Pittsburgh Pittsburgh, PA United States

**Keywords:** HIV, aging, MSM, gay and bisexual men

## Abstract

**Background:**

With the graying of sexual and gender minority communities and the growing number of people aged ≥50 years living with HIV, it is increasingly important to understand resilience in the context of the psychosocial aspects of aging and aging well.

**Objective:**

This paper aims to describe the methods and sample for the *Understanding Patterns of Healthy Aging Among Men Who Have Sex With Men* study*.*

**Methods:**

This observational cohort study was conducted within the Multisite AIDS Cohort Study (MACS) and was designed to explore resiliencies to explain patterns of health and illness among middle-aged and older sexual minority men. To be eligible, a participant had to be an active participant in the MACS, be at least 40 years of age as of April 1, 2016, and report any sex with another man since enrollment in the MACS.

**Results:**

Eligible participants (N=1318) completed six biannual surveys between April 2016 and April 2019. The mean age of the sample was 59.6 years (range 40-91 years). The sample was mostly White, educated, gay-identified, and included both HIV-positive (656/1318, 49.77%) and HIV-negative (662/1318, 50.23%) men.

**Conclusions:**

Understanding resiliencies in aging is a critical springboard for the development of more holistic public health theories and interventions that support healthy aging among older sexual minority men.

**International Registered Report Identifier (IRRID):**

RR1-10.2196/25750

## Introduction

### Background

Although difficult to accurately measure, it is estimated that there are currently 1.75 to 2.4 million sexual minorities (eg, lesbian, gay, and bisexual) in the United States who are aged ≥50 years; by 2030, it is estimated that there will be over 5 million sexual minorities [1]. Across the life course, many sexual minority men (SMM; including gay, bisexual, and other men who have sex with men) experience health disparities related to experienced stigma and discrimination, including increased depression or anxiety and substance use [2-7]. These disparities are often compounded by intersecting identities (eg, race or ethnicity and advanced age). The interactions of HIV (including long-term treatment, HIV infection, and HIV-associated non-AIDS conditions), health disparities, and aging—nearly half of persons living with HIV are aged ≥50 years, a majority of whom are SMM [8]—contribute to complex health conditions that create challenges to care and well-being [9,10].

The synergistic interplay of multiple psychosocial conditions that result in disparities in disease burden has been defined as a syndemic (ie, two or more conditions that interact synergistically to create excess disease burden) [11-15]. In a probability sample of urban SMM, Stall et al [15] were the first to show that a syndemic interplay of psychosocial health conditions (ie, depression, substance abuse, childhood sexual abuse, and violence victimization) was associated with HIV behavioral risk-taking and HIV infection among SMM. These initial findings have been replicated with many other independent samples of SMM in the United States and abroad and have been a bedrock in deficit-based theory and practice [16-28].

### Resiliencies: A New Approach

Despite these health disparities and exposure to stigma and discrimination, many SMM have managed to survive and thrive in the face of adversity. This study builds on the work of Fredriksen-Goldsen, Emlet, and others who have identified resiliencies in this population [29-32].

Theoretical definitions of resiliency focus on strengths in the face of adversity, suggesting that resilience is an ever-changing interaction of internal and external exposures to risk and protective factors and an individual’s adaptation to or recovery from adversity, rather than an innate or static trait [33-37]. Protective factors (ie, assets and resources) in the face of adversity are the foundation for the production of multiple resiliencies that men can draw upon to support health. Two primary resiliency models describing different pathways between protective factors and positive adaptation [33,36,38] have been identified. These are (1) the compensatory (main effects) model, in which the presence of resiliency factors has a direct positive effect on health outcomes, and (2) the protective (interaction) model, in which individual and environmental resiliency factors moderate the relationship between risks and health outcomes.

The development and expression of resiliencies in midlife SMM is still largely unknown. This study is designed to explore multiple factors that may work individually or in combination to act as protective factors that may be associated with better mental and physical health outcomes and moderate effects of syndemic psychosocial conditions on HIV-related health. Our expectation is that this sets the stage for a series of manuscripts that use these data to characterize resiliencies among SMM and may demonstrate protective effects on health.

Current risk-focused theoretical models, such as syndemics, have been unable to fully capture the complexities that underlie health disparities that burden SMM over the life course, including resiliencies that buffer or interact with the stressors that drive these disparities [39]. In creating a theoretical model inclusive of both risk and resilience, we may be able to identify pathways for innovative and practical methods for intervention, complementing the minimization of risk factors while strengthening health-promoting factors.

The study of resiliencies is relatively new to aging SMM health research; therefore, a community-engaged process was used to identify domains of focus [39]. Briefly, in 2011, members of the SMM community, researchers, providers, and other experts in sexual minority health were invited to a symposium to discuss how resiliencies might inform HIV prevention interventions. This group identified more than 200 SMM-specific resiliencies that were qualitatively collapsed and categorized. The symposium organizers then reviewed and amended these resiliencies with other theoretically important factors with a focus on identifying modifiable factors to create a list of factors that might be incorporated into future research efforts. The final list included resiliencies that operate at the level of the individual (eg, managing internalized homophobia and shame, self-monitoring and goal setting, adaptability, and coping), relationship building (eg, ability to form relationships and dyadic support), familial support (eg, building strong relationships with one’s family of origin and creating a family of one’s own), and structural and community support (eg, connection to community, institutional support, community building, homophobia management, and external monitoring) [39].

### Study Objectives

This paper describes the methods for and characterizes the sample of an observational cohort study designed to explore the production of resiliencies and to explain patterns of health and illness in aging among middle-aged and older HIV-positive and HIV-negative SMM. The overarching goals of the National Institutes of Health funded grant were to (1) identify individual interpersonal and structural resiliencies and evaluate their psychometric properties and determine their prevalence among middle-aged and aging SMM with and without HIV over time and (2) to investigate whether these resiliencies impact—separately and jointly—health and wellness outcomes, including virologic and immunologic control, depression, frailty, chronic disease (eg, diabetes and hypertension) management, and physical and cognitive functioning by mitigating the effect of psychosocial and behavioral vulnerabilities (eg, substance and alcohol use, partner violence, internalized homophobia, and social discrimination).

## Methods

### Study Design and Setting

This study, *Understanding Patterns of Healthy Aging Among Men Who Have Sex With Men* (“Healthy Aging Study,” NIMHD, R01M010680, principal investigator: RS, MRF, and MWP), is an observational cohort study conducted within the ongoing Multisite AIDS Cohort Study (MACS).

### The Multicenter AIDS Cohort Study

#### Overview

The MACS started enrollment in 1984 with sites located in Baltimore/Washington DC, Chicago, Pittsburgh, and Los Angeles [40]. In 1987, investigators from the Department of Epidemiology of the Johns Hopkins Bloomberg School of Public Health established the Center for Coordination, Analysis, and Management of the Multicenter AIDS Cohort Study (CAMACS). A total of 7352 men who had sex with men were enrolled in the MACS. In this prospective cohort study, HIV-positive and HIV-negative men were followed up every 6 months with interviews, physical examinations, cognitive testing, and phlebotomy. As one of the longest HIV cohort studies in the United States, the MACS has been able to provide a wealth of longitudinal biological and behavioral data on HIV risk prevention, seroconversion, disease progression or treatment, and quality of life. These factors made the MACS an ideal study to embed this study. More information can be found on the web [41].

#### Coordinating Center

The Healthy Aging Study Coordinating Center was a collaboration between the University of Pittsburgh School of Public Health and Georgetown University Medical Center. Working closely with CAMACS, the coordinating center was responsible for the oversight of the Healthy Aging Study, including training and working with the local study sites for participant recruitment and follow-up and data collection, data monitoring and safety, assuring communication between local sites, and monitoring the processes of data analysis and manuscript or conference abstract preparation.

#### Screening, Recruitment, and Enrollment

To be eligible for inclusion, participants had to (1) be an active participant in the MACS (attended at least one semiannual visit in the 2 years before the start of the Heathy Aging Study at MACS visit 65); (2) be at least 40 years of age at visit 65 (April 1, 2016); and (3) report any sex with another man since enrollment in the MACS. The initial eligibility list was updated before each of the subsequent five Healthy Aging Study visits to remove individuals who died, withdrew from the MACS, or requested to be removed from future recruitment. Although the total number of possible participants was set according to the MACS cohort at visit 65 (eg, no one was able to *age into* being eligible), men were able to enroll or choose not to participate in each of the six study visits.

Local site program staff recruited and enrolled all participants using the pre-existing MACS computerized-assisted direct interview (CADI) system. For each potential recruit, CAMACS first conducted an initial screening using historical MACS data. These data were used to program the CADI to alert the local study site staff of an eligible participant during their routine MACS visit. An initial CADI screen prompted the staff to ask eligible participants if they were interested in the study. If yes was entered, the following screen asked if they preferred to fill out a paper or electronic version of the survey. The same procedure was used for both in-person and phone visits. Participants were asked at each study from visit 65 through visit 70.

### Data Collection

#### Healthy Aging Study Surveys

Data collection for this study was completed within the existing structure of the ongoing MACS (ie, visit 65 of the MACS parent study was visit 1 for this substudy). This allowed us to decrease participant burden, as it did not require an additional study visit beyond MACS participation. Eligible participants were asked to complete a web-based or paper survey at each MACS visit between April 2016 and April 2019 for a total of six surveys, yielding a 3-year longitudinal assessment of both vulnerabilities and resiliencies. The periods for each visit were as follows: Healthy Aging Study visit 1-MACS visit 65: April 2, 2016-October 7, 2016; Healthy Aging Study visit 2-MACS visit 66: October 1, 2016-April 8, 2017; Healthy Aging Study visit 3-MACS visit 67: March 31, 2017-October 28, 2017; Healthy Aging Study visit 4-MACS visit 68: September 26, 2017-March 31, 2018; Healthy Aging Study visit 5-MACS visit 69: March 30, 2018-November 8, 2018; and Healthy Aging Study visit 1-MACS visit 70: October 2, 2018-April 8, 2019. Those who preferred an electronic survey were given the opportunity to complete the survey on an electronic tablet during or after their regular MACS visit or on a computer or personal device before or after their MACS visit using a link sent via email. Participants who did not want to complete the survey electronically were given the paper survey to complete after their MACS visit or to take home with an addressed stamped envelope for them to return to the study coordinating center. All surveys were completed within 1 month of the corresponding study visit. Paper surveys were sent to the coordinating center for entry into the study database. The survey was conducted in English and Spanish.

The coordination center provided a weekly tracking report of *Healthy Aging* statistics, including eligibility, enrollment, survey completion, and pending or outstanding survey completions to local MACS sites. Sites used these data to follow up with participants to remind and encourage survey completion using locally established and approved MACS follow-up procedures (eg, phone, email, and mail). The coordinating center worked with individual sites, as necessary, on issues related to follow-up. All surveys had to be completed within 1 month before or after the participant’s scheduled MACS visit. Surveys submitted after this window were censored.

#### Healthy Aging Study Measures

The surveys included a range of questions and scales to assess global resilience (Global Resiliency Scale- Resiliency Scale-14 [42]), theorized resiliencies (eg, social bonding using the Social Provisions Scale [43] and Relationships structures using the Experiences in Close Relationship-Resiliency Scale [44]), psychosocial and behavioral vulnerabilities (eg, loneliness using the University of California, Los Angeles Loneliness Scale [45] and alcohol use using the Alcohol Use Disorders Identification Test-Concise [46]), and socioeconomic descriptors (eg, sexual or gender identity, income, and employment). We included well-established validated items (eg, Alcohol Use Disorders Identification Test-Concise [46] and Global Resiliency Scale-Resiliency Scale-14 [42]); the study team designed items to assess more novel concepts for which established instruments did not already exist (eg, homophobia management) and some items were developed through community engagement (eg, working with Let’s Kick ASS to develop the AIDS survivor syndrome questions [47]).

Theorized resiliencies were chosen based on the domains identified through the community-engaged process described earlier. We intentionally chose measures to assess possible resiliencies at the individual, interpersonal or dyadic, and community levels. We also included several syndemic-informed measures at each level. These measures were primarily selected from previous studies [14,17,39].

To assess the widest range of resilience and syndemic factors while respecting survey length and participant burden, we chose to identify some measures to ask for each survey wave (eg, global resiliency and social bonding), whereas others were asked intermittently, for example, at two of the waves within the 3-year follow-up period (eg, homophobia management and physical activity). This syncopated process also allowed the study team to add additional factors that were not initially included in the first *Healthy Aging* survey (eg, conversion therapy and grit). The final visit 70 survey included nearly every question asked over the course of the study. Table 1 describes each of the study measures, at which visits participants were asked to provide an overview of data points for future longitudinal analyses, and how each measure is connected to the larger MACS.

**Table 1:** Measures of the Multisite AIDS Cohort Study Healthy Aging Study (2016-2019).

Domain or measure			Study visit^a^										
Aging visit			1		2		3		4		5		6
MACS^b^ visit			65		66		67		68		69		70
**Individual level resiliencies**													
	Housing status^c^	✓^d^		✓		✓		✓		✓		✓	
	Volunteer work: General Social Survey [48]	✓		✓		✓		✓		✓		✓	
	Global Resiliency Scale (RS-14^e^) [42]	✓		✓		✓		✓		✓		✓	
	Revised Life Orientation Test [49]	✓		✓		✓		✓		✓		✓	
	Multidimensional measurement of religiousness or spirituality [50,51]	✓		✓		✓		✓		✓		✓	
	Self-monitoring and goal setting^c^					✓				✓		✓	
	Homophobia management^c^			✓		✓				✓		✓	
	Aging Satisfaction: Attitudes Toward Aging Subscale from Philadelphia Geriatric Center Morale Scale [52]	✓		✓				✓				✓	
	Grit [53,54]									✓		✓	
	Medical decision making (adapted from Sudore et al [55])					✓				✓		✓	
	Mindfulness: MAAS^f^ [56]					✓				✓		✓	
	Sexual health: IIEF^g^ [57]							✓				✓	
	Physical activity: IPAQ-SF^h^ [58]	✓		✓		✓						✓	
	Body image: BIQ^i^ [59]			✓		✓						✓	
**Individual level other**													
	Internalized homophobia [60]	✓		✓		✓		✓				✓	
	Employment^c^					✓		✓		✓		✓	
	Work satisfaction: BIAJS^j^ [61]	✓		✓		✓		✓				✓	
	UCLA^k^ loneliness [45]	✓		✓		✓		✓				✓	
	Sexual orientation^c^	✓		✓		✓		✓		✓		✓	
	Gender identity^c^	✓		✓		✓							
	Sexual behavior with gender minority partners^c^	✓		✓		✓							
	Bisexual orientation and stigma^c^					✓						✓	
	Discrimination experiences^c^	✓		✓		✓		✓		✓		✓	
	Alcohol use: AUDIT-C^l^ [46]	✓		✓		✓		✓		✓		✓	
	Sex work^c^					✓						✓	
	Health care satisfaction: PSQ-18^m^ [62]	✓		✓		✓		✓		✓		✓	
	HIV biological prevention techniques^c^					✓				✓		✓	
	AIDS survivor syndrome^c^ [47]	✓		✓		✓		✓		✓		✓	
	Anxiety: GAD-7^n^ [63]	✓		✓		✓		✓		✓		✓	
	Pain experiences and treatment^c^									✓		✓	
	Opioid use^c^									✓		✓	
	Pill burden scale [64]									✓		✓	
	Posttraumatic stress disorder: PCL-C^o^ [65]							✓		✓		✓	
	Stigma experiences^c^							✓				✓	
	Digital communication use^c^							✓					
	Technology use^c^							✓					
	Conversion therapy experiences^c^			✓		✓						✓	
	Stress: PSS-10^p^ [66]									✓		✓	
**Interpersonal or dyadic level resiliencies**													
	Social bonding-Social Provisions Scale [43]	✓		✓		✓		✓		✓		✓	
	Support^c^	✓		✓		✓		✓		✓		✓	
	Mentoring: Gay Mentoring Scale [67]	✓		✓		✓		✓		✓		✓	
	Relationships^c^					✓		✓		✓		✓	
	Relationship structures-ECR-RS^q^ [44]			✓		✓		✓		✓		✓	
**Interpersonal or dyadic level other**													
	HIV status and HIV disclosure or stigma^c^	✓		✓		✓		✓				✓	
	IPV^r^ [68,69]									✓		✓	
**Community or structural level resiliencies**													
	Emotional connection with gay community [70]	✓		✓		✓		✓		✓		✓	
	Psychological Sense of Community [70]	✓		✓		✓		✓		✓		✓	
	Neighborhood contexts^c^			✓		✓				✓		✓	

^a^Sample size at each visit: visit 65: n=871; visit 66: n=1118; visit 67: n=1116; visit 68: n=1065; visit 69: n=1071; visit 70: n=1056.

^b^MACS: Multisite AIDS Cohort Study.

^c^Study team–developed measure.

^d^Measure present.

^e^RS-14: 14-item Resilience Scale.

^f^MAAS: Mindful Attention Awareness Scale.

^g^IIEF: International Index of Erectile Function.

^h^IPAQ-SF: International Physical Activity Questionnaire-Short Form.

^i^BIQ: Body Image Questionnaire.

^j^BIAJS: Brief Index of Job Satisfaction Measure.

^k^UCLA: University of California, Los Angeles.

^l^AUDIT-C: Alcohol Use Disorders Identification Test-Concise.

^m^PSQ: Patient Satisfaction Questionnaire.

^n^GAD-7: Generalized Anxiety Disorder-7 item.

^o^PCL-C: Posttraumatic stress disorder checklist.

^p^PSS-10: Perceived Stress Scale-10 item.

^q^ECR-RS: Experiences in Close Relationships-Relationships Structures.

^r^IPV: intimate partner violence.

#### Childhood, Coming Out, and Early Adulthood Survey

The *Long Term Health Effects of Methamphetamine Use in the MACS* (NIDA, R01DA022936, principal investigator: RS) study between 2008 and 2009 collected information on experiences related to childhood, coming out, and early adulthood [17]. Although some of the *Healthy Aging* Study participants completed this survey at that time, not everyone did. Healthy Aging Study participants who had not completed this survey in 2008-2009 were given the opportunity to do so at each study wave until completion or refusal. The baseline survey provides important additional data on participants’ experiences related to childhood, adolescence, and coming out. A total of 195 participants completed the survey.

### MACS Core Variables

#### Overview

*Healthy Aging* Study participants were matched by unique ID to the longitudinal MACS data, thereby connecting them to many years of psychosocial, behavioral, and biological data collected as part of their regular MACS participation. Data and specimens collected at semiannual visits included prospectively measured HIV status (for HIV-negative participants) demographic and psychosocial characteristics; medications used as pre-exposure prophylaxis or HIV treatment; hematologic variables, including an enumeration of CD3, CD4, and CD8 T-cell subsets; plasma HIV RNA quantification (for HIV-positive participants); a lipid profile; hepatitis serology; liver and renal function assays; evaluation of glucose metabolism and the allocation of samples for repository; HIV-related symptoms; psychomotor functioning; and illnesses and use of health services. Matching participants to these data is essential for completing the *Healthy Aging* Study objectives. This has provided an opportunity for study investigators to develop additional related projects.

#### Participant Incentives

Participants were reimbursed US $35 for each *Healthy Aging* survey for a possible total of US $210 over the entire study period, in addition to regular MACS participation incentives. For wave 70, to increase participation and adjust for longer lengths, reimbursement was increased to US $45. Participants who completed the *Childhood, Coming Out, and Early Adulthood* Survey during this time (ie, did not complete it in 2008-2009) were reimbursed an additional US $20. MACS staff issued all incentives according to the existing protocols. If not completed onsite, the Healthy Aging Study Coordinating Center notified the local MACS site when a survey was completed, prompting the site to issue the incentive.

#### Human Subjects and Informed Consent

All study procedures were approved by the coordinating center at the University of Pittsburgh. Subsequently, the approved protocol was submitted for approval by each of the participating MACS sites. Informed consent was obtained from all participants using a click-to-consent procedure at the beginning of each electronic survey at each study wave. Those who completed the hard copy surveys were asked to consent by checking a box. Some local sites also required a hard copy consent to be completed, which was obtained by the local study staff.

To strictly protect participants’ confidentiality, all study data were coded by the participant’s MACS ID. Any identifying information associated with this ID was kept at the local sites in accordance with the approved protocols. Number-coded information became part of an electronic database, which was password-protected and only accessible to the study staff.

### Analyses

Analyses included in this paper were completed in SPSS (version 26) software to characterize the sample at *baseline* (ie, the initial visit for each participant). Frequencies were used to describe the overall sample, with comparisons by HIV status using Pearson chi-square, Fisher exact, and two-tailed *t* tests as appropriate.

Previous publications from this study used several analytical approaches to assess research questions. These approaches include longitudinal latent class analyses to identify associations between social environmental resilience and loneliness [71] and associations between social support typologies and depression symptoms [72]; group-based trajectory approaches to assess predictors of polypharmacy [73]; longitudinal multinomial analyses assessing predictors of romantic partnership structures [74] and predictors of aging satisfaction [75]; multivariable regressions to assess the effects of conversion therapy on depressive symptoms [76]; longitudinal mixed models with lagged variables assessing the effects of employment status on depressive symptomology [77]; and structural equation modeling, using mediation, to assess associations among gay community connection, negative self-appraisal, and fitness engagement [78]. The core investigative team will use Longitudinal Latent Class Analysis and structural equation modeling procedures to assess latent resiliency phenotypes and their direct and indirect effects on biopsychosocial health outcomes in a forthcoming series of manuscripts.

## Results

### Enrollment and Survey Participation

**Figure 1:**
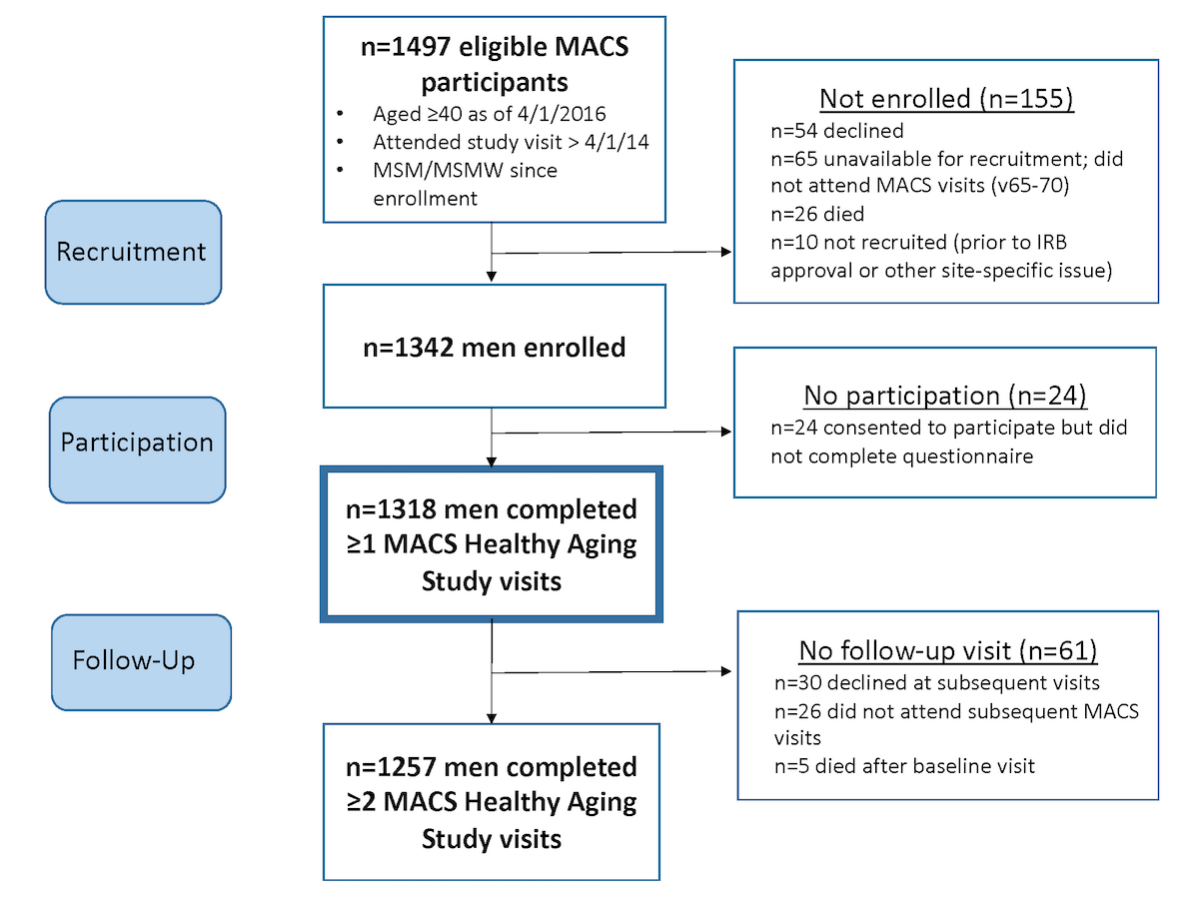
Healthy Aging Study participant recruitment and survey completion. IRB: institutional review board; MACS: Multisite AIDS Cohort Study; MSM: men who have sex with men; MSMW: men who have sex with men and women.

Figure 1 details the participant enrollment and follow-up for the *Healthy Aging* Study. There were 1497 MACS participants who met the eligibility criteria for the *Healthy Aging* Study at study commencement in April 2016. The target number changed as participants withdrew or passed away. Of the maximum 1497 potentially eligible MACS participants, we enrolled 1318 (88.04%) unique individuals into this study, who contributed a total of 6297 person-visits. Table 2 describes enrollment and participation numbers by visit. A mean of 1185 participants were approached across all waves ranging from 979 at visit 65 to 1199 at visit 70. The sample size was lower in visit 65 because some sites did not begin *Healthy Aging* enrollment until midway through the 6-month study visit period, due to delays in site-based institutional review board approval or logistics delays. Enrollment at visits were as follows: (1) visit 65: 91.6% (897/979); (2) visit 66: 89.93% (1152/1281); (3) visit 67: 86.47% (1120/1295); (4) visit 68: 91.7% (1127/1229); (5) visit 69: 92.16% (1129/1225); and (6) visit 70: 91.74% (1100/1199). The refusal across time was ranged from a high of 13.51% (175/1295) at visit 67 to a low of 7.84% (96/1225) at visit 69. Survey completion at visits were as follows: (1) visit 65: 97.1% (871/897); (2) visit 66: 97.05% (1118/1152); (3) visit 67: 99.64% (1116/1120); (4) visit 68: 94.23% (1062/1127); (5) visit 69: 94.86% (1071/1129); and (6) visit 70: 95.91% (1055/1100). Among Healthy Aging participants, 95.98% (1265/1318) completed two or more study visits, with a median completion of five visits (IQR 4-6).

Overall, just over half (3524/6293, 56%) of the surveys were completed at the clinic; the other 43.86% (2760/6293) were completed at home before or immediately following the study visit. Over time, the number of participants who completed the survey at home increased from 21% (183/871) at visit 65 to 60% (633/1055) at visit 70. When asked about the survey length, from 1=*too short* to 5=*too long*, the mean score across time was 3.7 (3=*just right*). When asked how *interesting* they found the surveys, participants reported an overall score of 3.6 on a scale from 1=very boring, 3=just okay, to 5=very interesting.

**Table 2:** Enrollment and participation for the Multisite AIDS Cohort Study Healthy Aging Study (2016-2019).

Visit	Targeted at visit^a^, n	Approached, n (%)	Refused, n (%)	Enrolled, n (%)	Complete surveys, n (%)	Incomplete surveys, n (%)
65	1497	979 (65.4)	82 (5.48)	897 (59.92)	871 (58.18)	26 (1.74)
66	1474	1281 (86.91)	129 (8.75)	1152 (78.15)	1118 (75.84)	34 (2.31)
67	1466	1295 (88.34)	175 (11.94)	1120 (76.4)	1116 (76.13)	4 (0.27)
68	1461	1229 (84.12)	102 (6.98)	1127 (77.14)	1062 (72.69)	65 (44.49)
69	1460	1225 (83.9)	96 (6.58)	1129	1071 (77.34)	58 (3.97)
70	1404	1199 (85.4)	99 (7.05)	1100 (78.35)	1055 (75.14)	45 (3.21)

^a^The targeted number changed from visit to visit due to death and withdrawals.

### Participant Demographics

Demographic and other descriptions of the sample are presented in Table 3. The mean age of the total sample was 59.6, ranging from 40 to 91. Most of the men were White (962/1318, 72.98%), Black (266/1318, 20.18%), or multiracial (44/1318, 3.34%) and 8.72% (115/1318) identified as Hispanic. Nearly everyone was identified as either gay (1152/1318, 87.41%) or bisexual (60/1318, 4.55%). Overall, the sample was highly educated, with 65.71% (866/1318) reporting a college degree (333/1318, 25.27%) or higher (533/1318, 40.44%); just under a third (41/1318, 3.11%) reported having less than a high school degree or General Educational Development. The sample was virtually equally divided between HIV-positive (656/1318, 49.77%) and HIV-negative (662/1318, 50.23%) participants. There were differences between these subsamples in terms of age, race, ethnicity, education, and sexual orientation. This sample also differs in some regard from those eligible MACS participants who did not enroll in the study (n=179; Multimedia Appendix 1). Overall, the Healthy Aging Study participants were on average younger (59.9 years compared with 62.2 years; *P*=.005) and among those living with HIV, had a higher mean CD4 count (697.9 compared with 623.9; *P*=.03).

**Table 3:** Participant characteristics of the Multisite AIDS Cohort Study Healthy Aging Study (2016-2019).

Characteristic		Total (N=1318)	HIV negative (n=662)	HIV positive (n=656)
**Age (years)^a^**				
	Value, mean (SD); range	59.8 (8.7); 40-91	62.1 (8.7); 41-91	57.4 (8.1); 40-82
	Value, median	60	62	57
**Race, n (%)^a^**				
	White	962 (73)	547 (82.6)	415 (63.3)
	Black	266 (20.2)	82 (12.4)	184 (28)
	Multiracial	44 (3.3)	13 (2)	31 (2.4)
	American Indian or Alaskan Native	14 (1.1)	5 (0.8)	9 (1.4)
	Asian	6 (0.5)	5 (0.8)	1 (0.2)
	Native Hawaiian or Pacific Islander	2 (0.2)	1 (0.2)	1 (0.2)
	Another race	23 (1.7)	9 (1.4)	14 (2.1)
	Missing	1 (0.1)	0 (0)	1 (0.2)
**Ethnicity, n (%)^a^**				
	Hispanic	115 (8.7)	628 (94.9)	575 (87.7)
	Non-Hispanic	1203 (91.3)	34 (5.1)	81 (12.3)
**Education, n (%)^a^**				
	Less than high school	41 (31.1)	15 (2.3)	26 (4)
	High school or GED^b^	126 (9.6)	45 (6.8)	81 (12.3)
	Some college	285 (21.6)	106 (16)	179 (27.3)
	College degree	333 (25.3)	175 (26.4)	158 (24.1)
	Graduate school or higher	533 (40.4)	321 (48.5)	212 (32.3)
**Sexual orientation, n (%)^c^**				
	Gay	1152 (87.4)	598 (90.3)	554 (84.5)
	Bisexual	60 (4.6)	21 (3.2)	39 (5.9)
	Straight or heterosexual	33 (2.5)	18 (2.7)	15 (2.3)
	Something else	24 (1.8)	7 (1.1)	17 (2.6)
	Unsure, prefer not to say, or N/A^d^	38 (2.9)	13 (2)	25 (3.8)
	Missing	11 (0.8)	5 (0.8)	6 (0.9)

^a^*P*>.001.

^b^GED: General Educational Development.

^c^*P*>.01.

^d^N/A: not applicable.

## Discussion

### Principal Findings

In this paper, we describe the methods and sample for an observational cohort study to understand the patterns of healthy aging. We successfully recruited, enrolled, and tracked 1318 midlife or SMM (1318/1497,

88.04% of the eligible men within the MACS) over a 3-year period (six total biannual surveys). The mean age of the sample was 59.6, including men aged 40-91 years. Overall, the sample was mostly White, educated, gay-identified, and included both HIV-positive (656/1318, 49.77%) and HIV-negative (662/1318, 50.23%) men.

More than two-thirds of the participants (914/1318, 69.34%) completed at least five of the six surveys. Participation in this new method of data collection for the MACS, including a hybrid of web-based and hard copy surveys, was high with nearly all men (95.98%, 1265/1318) completing at least two *Healthy Aging* survey across the six visits. Men found that these methods were acceptable with the uptake of web-based home-based surveys, increasing from 21% (183/871) to 60% (633/1055) at the final visit. This suggests the potential utility and importance of using early study visits to acclimate participants to the study and to increase buy-in for home-based data collection. This approach may decrease the cost of longitudinal data collection in similar cohort studies. Participants reported that the survey lengths were *just right* and that their interest level was *just okay*. The high level of survey completion and follow-up over six waves of data collection provide strong evidence to support the feasibility of these methods for data collection in the MACS and similar cohorts.

In this study, we conceived resilience as a myriad of multidimensional processes occurring at multiple levels over the life course. This is informed by and builds upon the important work of others [2,29-32,79]. We used a community-engaged process to develop domains of potential resiliencies specific to SMM at the individual, interpersonal or dyadic, and community levels.

### Strengths and Limitations

The methods described here, in particular the use of a staggered survey design, allowed us to collect longitudinal data on a wide range of topics without overburdening participants with exhaustive and overly repetitive surveys at each follow-up. The design also provided opportunities for flexibility and innovation and the possibility of responding to historical or individual-level changes or concerns. As an example, we were able to add a series of questions on how men use different methods of bio-behavioral HIV prevention techniques (eg, pre-exposure prophylaxis), which had greatly expanded during the study period. It also allowed junior investigators to include new constructs of relevance that were highly relevant but not included in the original survey (eg, conversion therapy [76]). This flexibility also resulted in our ability to expand beyond the original syndemic-framed aims, as proposed in the original proposal. In the years since the first and subsequent submissions, being funded, implementation, and now analyses and dissemination of findings, we have begun to place more emphasis on a resiliencies-based frame in this work.

The study was nested within the MACS, the longest running HIV cohort study in the United States, providing a well-established and finely tuned structure within which to implement this work. We were able to capitalize on participants’ pre-established MACS visits and the highly trained local study staff with longstanding relationships with the men, which undoubtedly helped with recruitment and follow-up. To some extent, this may limit the generalizability of our findings. These men, many of whom have been enrolled in the MACS for ≥30 years, may be more apt to participate and to continue through follow-up surveys. They may also be unique from men not already engaged in research, for example, with regard to syndemics (eg, substance use) or resiliencies (eg, altruism). Furthermore, while subsequent enrollment periods for the MACS cohorts used a stratified recruitment approach by HIV serostatus that attempted to recruit HIV-positive and HIV-negative SMM with similar baseline characteristics, we recognize that such designs are imperfect and that the stigma and isolation associated with HIV infection is specific to SMM living with HIV. For this reason, we recommend that analyses using these data are stratified by HIV status, so that the effects of protective factors are characterized independently in the context of HIV. Another limitation is that we experienced several unforeseen issues when implementing the first survey (visit 65); therefore, not all sites were able to enroll participants until later in the 6-month cycle, resulting in a smaller sample compared with other periods.

An important strength of this design is the ability to connect the resiliencies captured in this study to the wealth of MACS behavioral and biological data. This provides a unique opportunity to explore resiliencies using longitudinal data on myriad health indicators beyond HIV. For example, our work evaluated the role of psychosocial factors in buffering the development of incident frailty. Another area of research is investigating the roles of perceived health care quality and anticipated discrimination in health settings on the outcomes and equity of diabetes and hypertension control within the *Healthy Aging* cohort.

### Conclusions

With the graying of sexual and gender minority communities and the growing population of middle-aged and older adults living with HIV, it is increasingly important to understand the psychosocial aspects of aging and aging well. Currently, there are limited data on aging populations, particularly those that incorporate community-specific questions. Although an assessment of global resilience is important, these communities also have unique stories of survival, traditions, support, and needs (eg, the importance of created or chosen family) in relation to their lives as sexual minority persons and as persons affected by or living with HIV [80]. Opportunities to combine longitudinal psychosocial and biomedical or clinical data are rare. This study provides a foundation to address this gap by connecting innovative measures with decades of biomedical data. More information on the measures and using these data can be found on the web [81]. Understanding resiliencies in aging is a critical springboard for the development of more holistic public health theories and interventions that support healthy aging among older SMM**.**

## Appendix

Multimedia Appendix 1 Healthy Aging Study participants compared to all Multisite AIDS Cohort Study participants (data from Healthy Aging Study, 2016-2019).

## Abbreviations

CADI: computerized-assisted direct interview
CAMACS: Center for Coordination, Analysis, and Management of the Multicenter AIDS Cohort Study
CFAR: Center for AIDS Research
MACS: Multisite AIDS Cohort Study
SMM: sexual minority men
