# Supplementary material for: Understanding Patterns of Healthy Aging Among Men Who Have Sex With Men: Protocol for an Observational Cohort Study
Source: JMIR Res Protoc. 2021 Sep 23;10(9):e25750. doi: 10.2196/25750 (PMC8498890; doi:10.2196/25750)
Supplement: Multimedia Appendix 1 [file resprot_v10i9e25750_app1.docx]

**Multimedia Appendix 1**. Healthy Aging Study participants compared to all Multisite AIDS Cohort Study participants (data from Healthy Aging Study, 2016-2019).

| **Table 4: Healthy Aging Study Participants Compared to all MACS Participants, MACS Healthy Aging Study, 2016-2019** | | | |
| --- | --- | --- | --- |
| Factor | Eligible but not Enrolled MACS Participants  (n=179) | Enrolled Participants  (n=1387) | p-value |
| Age, median (IQR) | 62.2 (55.2, 68.7) | 59.9 (53.7, 65.7) | 0.005 |
| Race |  |  |  |
| White, non-Hispanic | 130 (72.6%) | 917 (69.6%) | 0.69 |
| White, Hispanic | 6 (3.4%) | 70 (5.3%) |  |
| Black, non-Hispanic | 36 (20.1%) | 262 (19.9%) |  |
| Black, Hispanic | 2 (1.1%) | 12 (0.9%) |  |
| Native American | 0 (0.0%) | 4 (0.3%) |  |
| Asian or Pacific Islander | 0 (0.0%) | 9 (0.7%) |  |
| Other | 0 (0.0%) | 11 (0.8%) |  |
| Other Hispanic | 5 (2.8%) | 33 (2.5%) |  |
| Education |  |  |  |
| High school or less | 21 (11.7%) | 194 (14.7%) | 0.15 |
| Some college, no  degree | 60 (33.5%) | 337 (25.6%) |  |
| Undergraduate degree | 42 (23.5%) | 339 (25.7%) |  |
| Graduate or Postgraduate | 56 (31.3%) | 448 (34.0%) |  |
| Years of MACS Data, median (IQR) | 31.3 (14.0, 31.6) | 31.2 (13.6, 31.6) | 0.057 |
| HIV Positive | 91 (50.8%) | 656 (49.8%) | 0.79 |
| Detectable HIV Viral Load | 13 (14.6%) | 117 (17.9%) | 0.44 |
| CD4 T Cell Count, mean (SD) | 623.9 (263.7) | 697.9 (306.0) | 0.030 |
| History of AIDS Diagnosis | 17 (18.7%) | 82 (12.5%) | 0.10 |
|  |  |  |  |
